# Defining the role of perineuronal nets in Alzheimer’s disease pathology

**DOI:** 10.1002/alz.089449

**Published:** 2025-01-03

**Authors:** Rocio A. Barahona, Giovanna Rubio Salgado, Aashna R. Kono‐Soosaipillai, Kim N. Green

**Affiliations:** ^1^ University of California, Irvine, Irvine, CA USA

## Abstract

**Background:**

Condensed extracellular matrix structures called perineuronal nets (PNNs) preferentially enwrap the soma and stabilize proximal synapses of parvalbumin‐expressing inhibitory neurons in the cortex, serving as a protective barrier against neurotoxins. While PNN structural integrity declines in the healthy aging brain, this reduction is exacerbated in Alzheimer’s disease (AD). In the 5xFAD mouse model of amyloidosis, the elimination of microglia prevents reductions in PNN, suggesting microglia are responsible for the over‐degradation of PNNs observed in AD. Additionally, microglial elimination before plaque onset impairs plaque formation, suggesting microglia are critical for plaque development. We observe that plaque formation is inhibited in areas of PNN deposition, and therefore postulate that PNNs are protective against plaque accumulation and synaptic/neuronal damage.

**Methods:**

Expression of aggrecan protein (ACAN) is required for PNN formation, therefore we crossed ACAN floxed mice with Nestin‐Cre and 5xFAD to selectively prevent PNN production from all neurons. Mouse brains were harvested for the following 4 groups: wild‐type (WT) control, WT PNN knockout (KO), 5xFAD control, and 5xFAD PNN KO at 4 months and 8 months of age with n = 4‐6 per sex per group. One hemisphere was processed for immunohistochemistry and the other hemisphere for spatial transcriptomics.

**Results:**

At the 8‐month timepoint, immunohistochemical quantification of AmyloGlo+ plaques shows a significant increase in both total plaque volume and total plaque number in the cortex of the 5xFAD PNN KO group compared to the 5xFAD control group. Notably, plaque pathology spread to the upper cortical layers normally occupied by PNNs. Additionally, we know that the microglial response to plaques is important for protecting against dystrophic neurites and preventing the spread of tau pathology. Therefore, we investigated the microglia and plaque interaction and found that the absence of PNNs impairs the microglial response to plaques, suggesting that the ECM composition is integral to proper microglial sensing and responses.

**Conclusions:**

Increases in plaque load and an impaired microglial response to plaques by 8 months in the PNN KO group suggest PNNs protect against plaque accumulation and appear to be essential for proper microglial response to plaques.

